# Introduction to clinical research based on modern epidemiology

**DOI:** 10.1007/s10157-020-01870-3

**Published:** 2020-03-24

**Authors:** Junichi Hoshino

**Affiliations:** Toranomon Hospital, Nephrology Center, 2-2-2, Toranomon, Minato-ku, Tokyo, 105-8470 Japan

**Keywords:** Propensity score, Instrumental variables, Bootstrapping, Competing risk, Multiple imputation, Intractable disease, Modern epidemiology

## Abstract

Over the past 20 years, recent advances in science technologies have dramatically changed the styles of clinical research. Currently, it has become more popular to use recent modern epidemiological techniques, such as propensity score, instrumental variable, competing risks, marginal structural modeling, mixed effects modeling, bootstrapping, and missing data analyses, than before. These advanced techniques, also known as modern epidemiology, may be strong tools for performing good clinical research, especially in large-scale observational studies, along with relevant research questions, good databases, and the passion of researchers. However, to use these methods effectively, we need to understand the basic assumptions behind them. Here, I will briefly introduce the concepts of these techniques and their implementation. In addition, I would like to emphasize that various types of clinical studies, not only large database studies but also small studies on rare and intractable diseases, are equally important because clinicians always do their best to take care of many kinds of patients who suffer from various kidney diseases and this is our most important mission.

## Introduction

After the development of the concept of evidence-based medicine (EBM), which has been in widespread use since the 1990 [1], many kinds of evidence-based clinical guidelines have been published. Thanks to advances in computer technologies and internet access, clinicians can now easily view these guidelines and apply the updated knowledge, which largely contributes to the improvement in the quality of care and uniformity of medical services provided. On the other hand, it also reveals the difficulty of implementing EBM for intractable diseases, rare diseases, and chronic diseases with a long history of progression because these diseases are often difficult to show sufficient evidence with “hard” outcomes based on “high-quality interventional clinical trials”. In nephrology, it is often difficult to show sufficient evidence for many diseases, and the US government recently started to approve of the use of an eGFR decline of 30% as a proxy outcome in clinical trials [2]. In addition, because of the very strict inclusion criteria of interventional trials, the implementation of the evidence written in the guidelines is often limited in nephrology clinical practice.

**Fig. 1:**
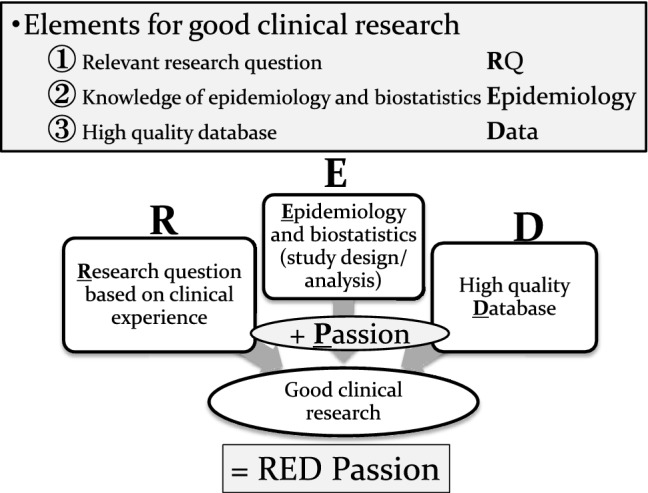
Elements for good clinical research

The mission of clinicians is to provide the best daily clinical practice based on updated clinical knowledge, which is improving daily. If evidence is not sufficient for bedside clinical practice, we have to solve clinical questions by ourselves using “real-world” clinical data. In general, evidence levels of observational studies are considered lower than those of interventional studies because of the existence of many biases: selection bias, information bias, missing data, publication bias, etc. However, recent advances in epidemiology (also known as modern epidemiology), information technology, and computer technology allow us to conduct high-quality observational studies. Advances in computer and internet technologies have been tremendous. Now, submitting a manuscript with analog photography by international mail has become a folk tale. It has been only 20 years since the first iMac by Apple Inc. and only 15 years since the release of Gmail by Google. Advances in these technologies have dramatically changed not only human life but also clinical research. In basic science, many advanced technologies have been launched, such as mass spectrometry, whole-genome sequencing, omics analyses, and single-cell analyses. These advanced technologies have dramatically changed many fields of medical sciences not only in basic science but also in clinical science. Thanks to these advances, all clinical researchers can perform advanced epidemiological and statistical techniques with their own personal computer, and clinicians have become more familiar with observational studies. However, statistical techniques are only one element for good clinical research. The other three elements are relevant research questions based on clinical experience, high-quality databases, and sufficient passion to accomplish clinical studies. These elements are equally essential for good clinical research (Fig. 1).

Here, I would like to introduce some fundamentals of clinical research and recent advances in the field of epidemiology.

## Fundamentals of clinical research

**Fig. 2:**
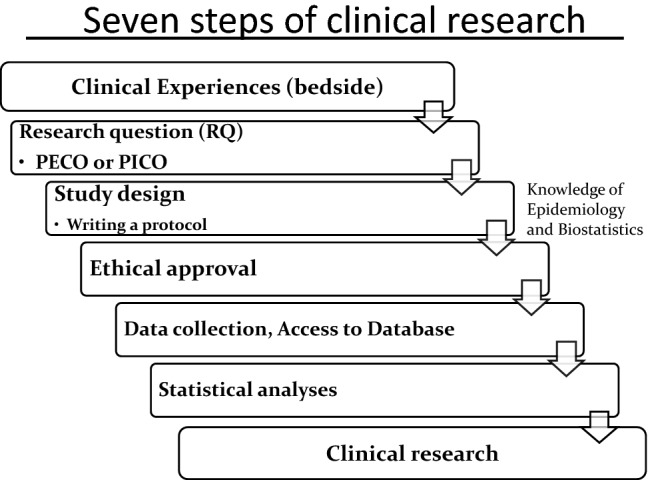
Seven steps of clinical research. *PECO* population, exposure, comparison, and outcome; *PICO* population, intervention, comparison, and outcome

As shown in Fig. 2, many clinical studies begin with clinical questions at the bedside. These questions are structured through the PE(I)CO format. It is essential to clarify definitions of the target population (P), exposures (E) or interventions (I), comparisons (C), and outcomes (O) as a first step in clinical research. All clinical research is conducted in accordance with the ethics guidelines for medical research for human subjects. Therefore, almost all clinical research should be started only after the approval of the ethics review committee. A good database (DB) is one that anyone can understand and reanalyze. Therefore, a codebook—a list of codes for each variable—and the updated version history should be stored together with the DB. In addition, it is also important to create an “alive” DB that is formatted in consideration of later statistical processing, rather than a “dead” DB that contains a mix of text and numerical characters. These “alive” DBs make it easy to use analytic software in the analysis process. DB creation and DB cleaning are time-consuming and labor-intensive tasks, but researchers should keep in mind that high-quality, traceable DBs are necessary for good clinical research.

We need to understand the strengths and weaknesses of large DBs. It is generally considered that a large, multicenter DB is the best because of its statistical power and low selection bias. However, large DBs often has difficulty obtaining additional data that researchers are interested in, and data access is often limited. Moreover, these larger DBs may contain more missing data and outliers than in-house data in general. Therefore, researchers should understand the mechanism of missing data and how to handle missing data and outliers before analyzing the data.

**Fig. 3:**
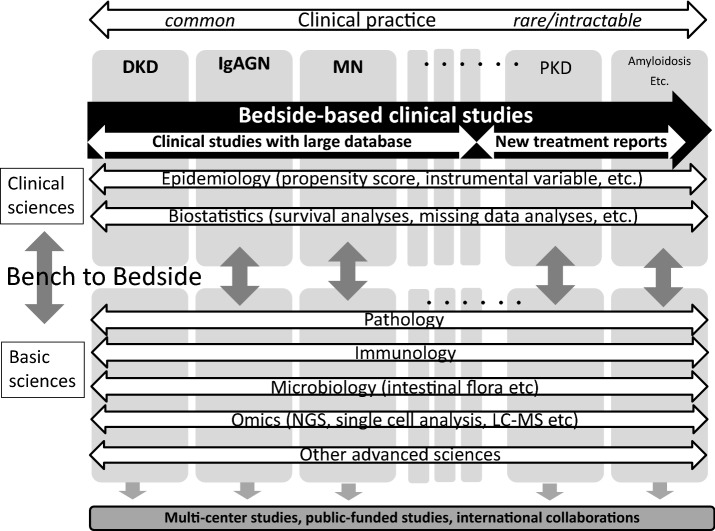
Associations between areas of kidney diseases and research technologies in clinical studies. *DKD* diabetic kidney disease, *IgAGN* IgA nephropathy, *MN* membranous nephropathy, *PKD* polycystic kidney disease

“Bench to bedside” has been an important concept for our medical researchers, since all advanced basic sciences are developed with the aim of treating patients. However, this concept may lead researchers to focus on one specific disease, since it may be the shortest route for researchers’ success to focus on one specific disease and to deepen their knowledge. If we consider the association between areas of kidney diseases and research technologies, advanced research techniques such as genome sequencing, omics analysis, single-cell analysis, and epigenomics can be applicable to many kinds of diseases and thus could be considered the horizontal axis (Fig. 3). Modern epidemiology could also be on the horizontal axis in the clinical research field. The combination of these horizontal technologies are essential to deepen knowledge. On the other hand, it is often difficult for clinical researchers to limit their areas of interest on the vertical axis because doctors cannot select patients to care for in clinical practice. In addition to these elements, the types of clinical studies may vary depending on the number of patients available. For common diseases, a large DB and knowledge of epidemiology and biostatistics may be essential for good clinical research. However, for rare diseases, papers on new treatments or even case reports are equally important for patients. These studies often do not require sophisticated statistical techniques. Therefore, we need to understand that the necessity of knowledge of epidemiology and biostatistics may depend on the commonality of a disease, and it may be natural for clinical researchers to have an interest in various kidney diseases and publish “bedside-based” clinical studies.

## Missing data and missing data analysis

Missing data not only leads to biased results but also potentially loses many observations, which reduces the power of the analysis. There are four famous approaches to handle the problem of missing data: (1) complete case analysis, (2) imputation, (3) weighting methods, and (4) model-based approaches. A complete case analysis is often the default of statistical software, but it may cause biased results and the power of the analysis will be reduced, as mentioned above. Commonly used imputation procedures include hot-deck imputation, mean imputation, and regression imputation, where the missing values are estimated by known variables. Multiple imputation may be the most popular method for imputation. It refers to the procedures of replacing each missing value by a vector of *D* ≧ 2 imputed values. The *D* values are ordered in the sense that *D* complete datasets can be created from the vectors of imputations; replacing each missing value by the first component in its vector of imputations creates the first completed dataset, replacing each missing value by the second component in its vector creates the second completed dataset, and so on. The model-based procedure defines a model for the observed data and bases inferences on the likelihood or posterior distribution under that model, with the parameters estimated by procedures such as maximum likelihood [3]. In addition, a mixed effects model may be an option to handle missing data. See Little’s textbook or other papers for more information [3, 4].

## Modern epidemiology and clinical research

Biases have always been a major problem for clinical researchers. Randomization is an excellent research design that can control all biases, including unknown biases. However, ethical issues, high costs, lack of external validity, and “uncontrolled” patients have been major limitations of this study design. On the other hand, because observational studies lack randomized assignment of participants into treatment conditions, researchers must employ statistical procedures to balance the data before assessing the treatment effects. In recent years, many excellent methods to control these biases have been developed, such as propensity score analysis, inverse probability weighting, marginal structural modeling, bootstrapping, instrumental variable, etc. [5–8]. Given the low cost and easy accessibility of DBs and the necessity of bias control, observational studies are considered an excellent tool for new researchers.

**Fig. 4:**
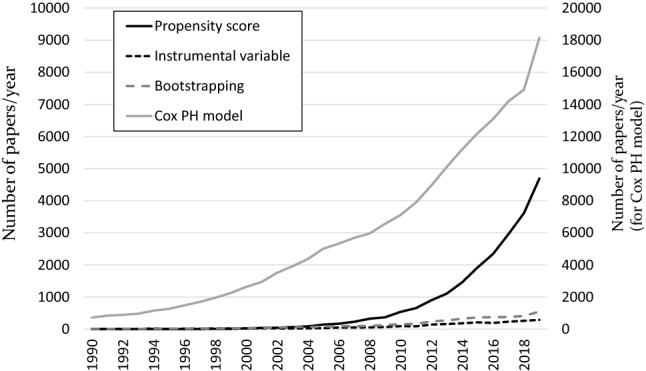
The number of published papers using modern epidemiological techniques. Cox PH model: Cox’s proportional hazard model. The number of published papers was calculated by PubMed on Feb 1, 2020

As shown in Fig. 4, the number of clinical papers using these modern epidemiological techniques has increased dramatically over the past couple of decades. It may be possible that these modern techniques will become familiar to all clinical researchers—such as Cox proportional hazards modeling in survival analysis—in the near future. Before implementation, we must keep in mind that there are some basic assumptions in a regression analysis: normally distributed errors (with a mean of zero), the independence of covariates (no multicollinearity), no correlation between the residual terms (autocorrelation), and homoscedasticity of the errors (equal variance around the line).

## Propensity score analyses

**Fig. 5:**
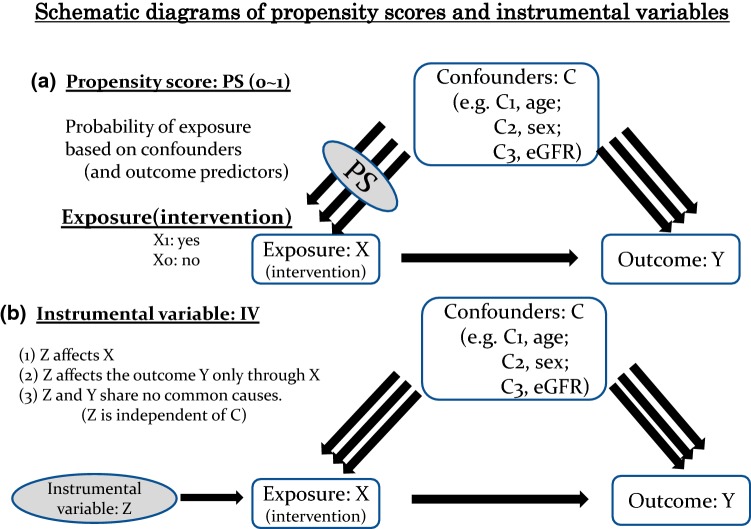
Schematic diagrams of propensity scores and instrumental variables. *eGFR* estimated glomerular filtration rate

In observational studies, treated and untreated subjects often differ systematically on prognostic factors leading to treatment selection bias or confounding in estimating the effect of a treatment on an outcome. Over the past 40 years, researchers have recognized the need to develop more efficient approaches for assessing treatment effects from observational studies, and statisticians (e.g., Rosenbaum & Rubin) and econometricians (e.g., Heckman) have developed a new approach called propensity score analysis [9–11]. A propensity score (PS) is the probability being assigned a treatment (or exposure) to accomplish data balancing when treatment assignment is unignorable, to evaluate treatment effects using non-experimental approaches and to reduce multidimensional covariates into a one-dimensional score (Fig. 5a).

There are five steps in a propensity score analysis: (1) selecting the variables for the PS model, (2) estimating the PSs, (3) applying the PS methods, (4) assessing the balance, and (5) estimating the treatment effect [12]. The PSs are calculated by logistic or probit regression models consisting of all the confounders in the given analysis set ranging from 0 to 1. In addition to all confounders, outcome predictors irrespective of the exposure can improve the precision of an estimated treatment effect without increasing the bias [12, 13]. Note that variables that are strongly related to the treatment but not to the outcome (instrumental variables) or weakly related to the outcome should not be included in the PS model because such variables could amplify the bias in the presence of unmeasured confounding [12–15]. Assuming that there are no unmeasured confounders, treated and untreated subjects with the same PS tend to have similar distributions of measured confounders [16]. For PS estimation, the c-statistics is often cited as a measure of “fit” of a PS model; that is, the ability of the model to predict treatment assignment using observed covariates [17–19]. The c-statistics takes on values between 0.5 (classification no better than a coin flip) to 1.0 (perfect classification). A very high or very low c-statistics implies the reduced utility of the PS approach. However, we have to keep in mind that the purpose of PS estimation is to balance risk factors for the outcome between treatment groups to eliminate confounding. Reliance on the c-statistics in selecting a PS model may provide false confidence that all confounders have been balanced between treatment groups [17]. For balance assessments, the standardized difference (SDif) is often used to assess the balance between the selected treated and untreated subjects. It is preferable that the SDif be less than 0.1 [20]. If not, changing the caliper of the PSs (usually 20% of the standard deviation of the PSs), selecting other matching methods, changing the replacement status of the matching selection, or rechecking the choice of variables for the PS model (e.g., checking interactions and linearity) are often performed. Recently, post-matching the c-statistics of the PS model has been suggested as an overall measure of the balance across covariates [21].

**Fig. 6:**
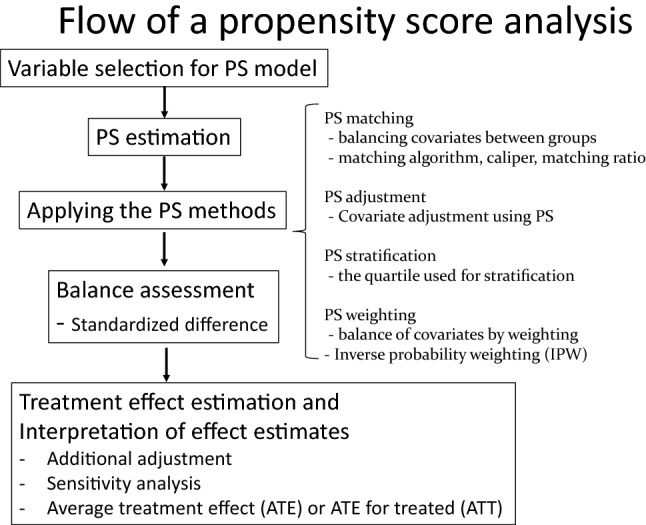
Flow of a propensity score analysis. *PS* propensity score

There are four major analytic approaches that use PSs: PS matching, PS adjustment, PS stratification, and PS weighting (Fig. 6). PS matching is the most widely used and probably most understandable PS analytical method [12]. On the other hand, because this method selects only matched subjects, it may result in an undesirable loss of study participants. Note that the exclusion of unmatched subjects from the analysis not only affects the precision of the effect estimate but also has consequences on the generalizability of the results. The other three methods—PS adjustment, PS stratification, and PS weighting—do not need to consider the loss of study participants. PS weighting aims to reweight the treated and untreated subjects to make them more representative of the population of interest without the loss of participants. Inverse probability weighting (IPW) using PSs has recently become popular. In IPW, the use of stabilizing weights could help “normalize” the range of the inverse probabilities and increase the efficiency of the analysis [12]. However, we should keep in mind that IPW tends to overweight participants with extremely small (or large) PSs. Freedman and Berk reported that PS weighting was optimal only under three circumstances: (1) when the study subjects are independently and identically distributed, (2) when selection is exogenous, and (3) when the selection equation is properly specified [22]. For treatment effect estimation, careful interpretation of the treatment effect estimate is needed. PS matching typically focuses on the effect of the treatment in either the treated or the untreated subjects, not on the average treatment effect on the whole population [23, 24]. PS adjustment and PS stratification give conditional treatment effect estimates [25]. However, marginal structural modeling methods using IPW estimate a marginal treatment effect in randomized studies; thus, the estimate can be directly interpreted as the average causal treatment effect between treated and untreated patients [26, 27]. See the textbook and papers on PS for more information [9, 12, 28, 29].

## Longitudinal data analyses

Survival analysis is one of the most familiar analyses for medical scientists. On the other hand, survival data have two common features that are difficult to handle with conventional statistical methods: censoring and time-dependent covariates. Kaplan–Meier analysis and Cox proportional hazard modeling are probably the most widely used techniques in our field (Fig. 4), although there are many different methods, including exponential regression, log-normal regression, Weibull AFT modeling, competing risks models, and discrete-time methods. Cox proportional hazards models have a basic assumption called the proportional hazards assumption: the hazard ratio comparing two groups is constant. This assumption is often tested by log–log plots and Schoenfeld residuals, which should be tested before estimation.

Additionally, competing risks are an important concept in clinical research, since survival analysis is often applied to study death or other events of interest. However, an important assumption of standard survival analyses such as the Kaplan–Meier method is that censoring is “independent”, which implies that the censored patients at a certain time point should be representative of those still at risk at same time. This condition is usually the case when a patient is lost to follow-up. In oncology and cardiovascular medicine, the analytical problem of competing risks has been acknowledged for many years. In nephrology, death and ESRD are competing risks in ESRD risk studies because death before ESRD prevents ESRD from occurring. If death before ESRD was censored, it may result in biased estimates because death is strongly related to future ESRD events [30, 31]. The cause-specific Cox hazard model is often suitable for studying associations between covariates and the instantaneous risk of a clinical event. However, the cause-specific hazard model cannot produce predicted probabilities of the event of interest without additional models for the competing risk event, because the predicted probability of ESRD must take into account both the incidence of ESRD and death. In this case, the sub-distribution hazard (SHR) approach proposed by Fine and Gray [32] is considered a preferable approach for prognostic studies, because this method can be used to predict the future risk of ESRD, taking into account the attrition due to death [5, 30]. Note that the SHR model directly provides individual probabilities of an event, given a patient’s characteristics, but it cannot be interpreted as a hazard ratio (HR) [6].

Qualifying the effect of treatment duration in survival analysis is a major problem for researchers, since only people who survive for a long time can receive a treatment for a long time. A direct comparison of subjects with longer and shorter follow-ups would be biased. For analyses of these longitudinal data with time-varying confounders, Robins reported a *g* formula to measure the healthy worker survivor effect [7]. Although this formula is complicated, Hernan recently reported a simple three-step approach to estimate the effect of treatment duration on survival outcomes using observational data [8]. The first step is duplicating people to assign them to treatment duration strategies at time zero, eliminating immortal time bias [33–35]. The second step is censoring the duplicates when they deviate from their assigned treatment strategies through follow-up. The introduced selection bias can be eliminated by the third step with inverse probability weighting to adjust for the potential selection bias introduced by censoring. Applications of these new approaches can be found in the areas of nephrology [36–38], infectious diseases [39, 40], gastroenterology [34], and urology [41].

## Instrumental variable method

Instrumental variable (IV) analysis is one of the methods used to control for confounding and measurement error in observational studies so that causal inferences can be made. This method was invented in the 1920s in economics and has appeared in the health sciences [42]. Suppose X and Y are the exposure and outcome of interest, respectively, and we can observe their relation to a third variable Z (Fig. 5b). Let Z be associated with X but not associated with Y except through its association with Y. Here, Z is called an IV [33]. That is, an IV is a factor associated with the exposure but not with the outcome. For example, the price of alcohol can affect the likelihood of expectant mothers drinking alcohol, but there is no reason to believe that it directly affects the child’s birth-weight [43]. There are three assumptions of IV: (1) Z affects X, (2) Z affects the outcome Y only through X, and (3) Z and Y share no common cause [42, 44]. The obvious example of an IV is in randomized controlled trials, since the random treatment assignment Z is independent of confounders and affects Y only through X. IV analyses are promising for the estimation of therapeutic effects from observational data as they can circumvent unmeasured confounding [45]. However, even if the IV assumption holds, Boef et al. reported that IV analysis will not necessarily provide an estimate closer to the true effect than conventional analyses, as this result depends on the estimates’ bias and variance [46]. They also reported that IV methods have the most value in large studies if considerable unmeasured confounding is likely and a strong and plausible instrument is available.

## Bootstrapping

In clinical research, we always assume that results from a sample population (e.g., CKD patients in our hospital) imply the same results in the source population (e.g., CKD patients in Japan or around the world). Given the central limit theorem and law of large numbers, the mean of all the samples from the population will be approximately equal to the mean of the population if the samples are large enough. Furthermore, all the samples will follow an approximate normal distribution, with all variances being approximately equal to the variance in the population, divided by each sample’s size. The original concept of bootstrapping was proposed by Bradley Efron [47, 48]*.* The basic idea of bootstrapping is that inference about a population from sample data can be modeled by resampling the sample data with replacement and performing inference about a sample from the resampled data. For example, after generating sufficiently large randomly selected pseudo-sample sets (e.g., 500 bootstrapping sample sets) from the original sample, the distribution of these statistics across the bootstrapping sample sets (e.g., the mean of the distribution of the means of bootstrapping sample sets) will be approximately equal to the distribution of the original sample statistics (e.g., the mean of the distribution of the original sample). This method is very useful when inferring the unknown distribution of the original sample statistics. This kind of simulation method has been developed and implemented in medicine [47, 49–51].

## Challenges for intractable kidney disease: importance, relevance and novelty

The treatment of intractable diseases requires a great deal of effort and passion, but from the viewpoint of clinical research, these diseases are often rare, and small studies, including case series papers, are valuable for bedside treatment. For example, clinical studies of pediatric nephritic syndrome and amyloidosis have been published in major journals despite the limited number of participants [52, 53]. These examples suggest that not only methodology and DB issues but also clinical relevance and novelty are very important in clinical research. For example, in 1996, we started using arterial embolization therapy for intractable PKD patients on dialysis [54]. This new treatment method can dramatically reduce the size of enlarged kidneys and improve their symptoms as well as nutritional conditions, and this method has spread across the world. Additionally, although diabetic nephropathy is a common disease, its pathophysiology is largely unclear. Therefore, we examined the association between pathological changes in diabetic nephropathy and clinical outcomes [55–62]. Other examples can be found in many areas, such as in the areas of treatment of polycystic liver disease [63, 64], amyloidosis [65–68], and severe ischemic limb treatment [69–72]. Clinical relevance and novelty sometimes overcome the limitation of the number of patients, especially in the areas of rare and intractable diseases.

## Closing remarks

Here, I briefly introduce modern epidemiology techniques, as well as the importance of novelty and relevance in all research areas of interest. PS analyses, IV methods, competing risk analysis, and other recently developed methods are becoming more familiar to clinical researchers. Thanks to recent advances in the field of computer science and internet technologies, it is now very easy to learn and perform these techniques by ourselves. On the other hand, there are many unsolved research questions, especially in the areas of rare diseases and intractable diseases. In these areas, it is not always necessary to use these modern techniques; rather, it is important to treat patients with sincerity and with passion. A case report or case series should be valuable in these areas. I hope that many doctors will become interested in clinical research, publish many excellent clinical papers, and as a result, contribute to improving patients’ quality of life.
